# Nesting Ecology of *Lepidochelys olivacea* in Lobito, Angola

**DOI:** 10.3390/mps7010002

**Published:** 2023-12-27

**Authors:** Inês M. Ferreira, Luz Murillo, Jean-Marie Le-Corre, Marco Correia, Rita Anastácio, Mário J. Pereira

**Affiliations:** 1 Department of Biology, University of Aveiro, 3800-193 Aveiro, Portugal; 2 Guardiões da Costa Mwangolé Association, Benguela, Angola; 3 Independent Researcher, 3800 Aveiro, Portugal; 4 Department of Biology & CESAM (Centre for Environmental and Marine Studies), University of Aveiro, 3800-193 Aveiro, Portugal

**Keywords:** Olive Ridley, Angola, east Atlantic Ocean, conservation, methodologies, female sizes, nesting success, endangered species, community involvement, citizen science

## Abstract

The scarcity on the Atlantic coast of the African sea turtle population and its dynamics data is well known. This article discusses the nesting ecology methods and analysis of a nascent Angolan project aimed at preserving the nesting female population of the Olive Ridley turtle (*Lepidochelys olivacea*) on the coast of Lobito. This study examines the nesting ecology of this species from 2020 to 2023. Females had an average CCL of 70.2 cm and CCW of 68.5 cm. These females laid 127 eggs in nests that averaged 47.0 cm deep. The *ex situ* nest incubation period averaged 60 days, and the hatchling success was 82.1%. Some techniques used in this project require modifications and enhancements. The utilization of photo identification did not yield the anticipated outcomes, prompting the adoption of passive integrated transponders (PITs) in the last season. However, due to limited funding, the success of this method is contingent upon an augmented field effort, allowing for the recapture of a larger number of females. The continuity of this project hinges upon collaboration between higher authorities and the local community. Together, it is possible to deepen the understanding of the nesting ecology of this species and address pivotal issues for its conservation, thereby implementing the most effective preservation measures.

## 1. Introduction

Sea turtles are a crucial part of marine ecosystems and play a vital role in maintaining their balance [1], serving as indicators of the overall state of these ecosystems [2]. In addition, these charismatic species support tourism contributing to local communities’ economies [3,4]. Besides these advantages, the threats faced by sea turtles, specifically by nesting populations, are also well known [5] and include habitat destruction and loss, bycatch, pollution, and climate change [6,7,8], which are mainly threats resulting from human activity. Knowing the size of populations, their trends, their genetic diversity, and their adaptability to global changes is crucial for debating mitigation strategies [9]. Currently, the widely used conservation measures are limited to protecting nesting sites, reducing pollution, and reducing bycatch and poaching [10]. One major obstacle many projects face is the lack of published data concerning nesting and feeding areas; for example, despite significant efforts in recent years, the Atlantic African coast still poses a major challenge to the conservation of sea turtle species [9,11], and finding scientific articles or reports is difficult.

According to the IUCN Red List, most species of marine turtles are in decline [12]. The Olive Ridley turtle (*Lepidochelys olivacea*) can be found in warm waters, including the Pacific, Atlantic, and Indian Oceans [13], and it has distinct characteristics such as its small size and the arribadas, a behavior known only in Olive Ridleys and Kemp’s Ridleys that is not observed at all nesting beaches [14,15,16]. The largest colonies of this species are found at La Escobilla Beach, Mexico, with approximately 800,000 nesting females per season [17], in India at Gahirmatha Beach, with 700,000 nesting females per year [18] and at Rushikulya Beach and Ostional Beach with 600,000 nesting females [19], and in Costa Rica with 200,000 nesting females in a single arribada [20].

The Olive Ridley turtle has a widespread range in west African coastal waters. Carr [21] identified the northern limit of its range in Mauritania as Dakhlet Nouadhibou (previously Lévrier Bay and Port Etienne). Carapaces discovered by Arvy and Dia [22], as well as recent specimens south of Nouakchott reported by Lematt et al. [23] based on Fretey’s findings, support its existence in Mauritania’s coastal region. The northern extent of its nesting range is marked by the Langue de Barbarie National Park in Senegal, near the Mauritanian border, as supported by a nest spotted in June 2011, as reported by Fretey [24]. As described by Carty et al. [25], Guinea-Bissau reports Olive Ridley nesting in the Bijagos Archipelago, with up to 620 nests estimated between 1992 and 1993 in Orango National Park alone. Fretey and Malaussena [26] and Siaffa [27] have observed females nesting on the beaches of Freetown, Baki, and the Sherbro Islands in Sierra Leone. Fretey et al. [28] claimed that the southern beaches of Liberia may potentially provide indications of Olive Ridley nesting. In the study conducted by Gómez et al. [29] and Gómez [30], it was found that a total of 149 Olive Ridley nests were documented in the vicinity of the Liberia–Ivory Coast border during the year 2001. However, the lack of viable data and comprehensive studies along this coastline still does not allow for a rigorous assessment of the population dynamics along the coast.

Of the five species reported to occur in Angola’s coast in the past [31,32], only three (olive, green, and leatherback turtles) species are reported to occur and nest in Angola [33]. The known projects either have not made the information available or are too recent and, as such, have insufficient information to address at least local knowledge deficiencies, which are fundamental for defining long-term and regional conservation strategies [11,34,35]. 

In recent years, the presence of Olive Ridley turtles along the coast of Angola, specifically between Soyo and Cuio, has been documented. A study published in 2007 and another two in 2022 shed light on this [15,33]. Cunha and colleagues [36] conducted the only genetic diversity study of a nesting population in the Palmeirinhas area (Luanda), providing crucial information for refining the definition of regional management units (RMUs). The coast of Angola falls under the east Atlantic RMU for *L. olivacea*, as determined by Wallace and colleagues [37]. However, the analysis of information considered by Wallace et al. [37] reveals that the definition of RMUs for the Angolan coast was based on a single descriptor, namely the nesting ecology, and in some cases, these data are over ten years old. Therefore, the lack of current knowledge raises several concerns about the effectiveness of conservation policies and measures [9,11]. This lack of information presents a challenge for new conservation projects that rely on current information and guidelines.

To address and contradict issues like the decreasing trend of the species in the Atlantic African coast or the lack of solid data to define RMUs, projects focused on conservation have been created, and many have been initiated by common citizens. This is the case of the “Cambeú Project”—Cambeú means “little turtle” in Umbundu (a local dialect)—a small marine turtle conservation project included in the Guardiões da Costa Mwangolé, a non-governmental organization in Angola. The project was founded in 2017 by two common citizens who recognized the threats faced by turtles, especially Olive Ridley, that were nesting right in front of their house. They started protecting the nesting females and their nests, and the project got bigger.

This article aims to provide information on the methodologies used and to analyze the nesting ecology, presenting the results obtained during the nesting seasons in 2020–2023 from the Cambeú Project at Lobito, Angola.

## 2. Materials and Methods

### 2.1. Study Area

**Figure 1:**
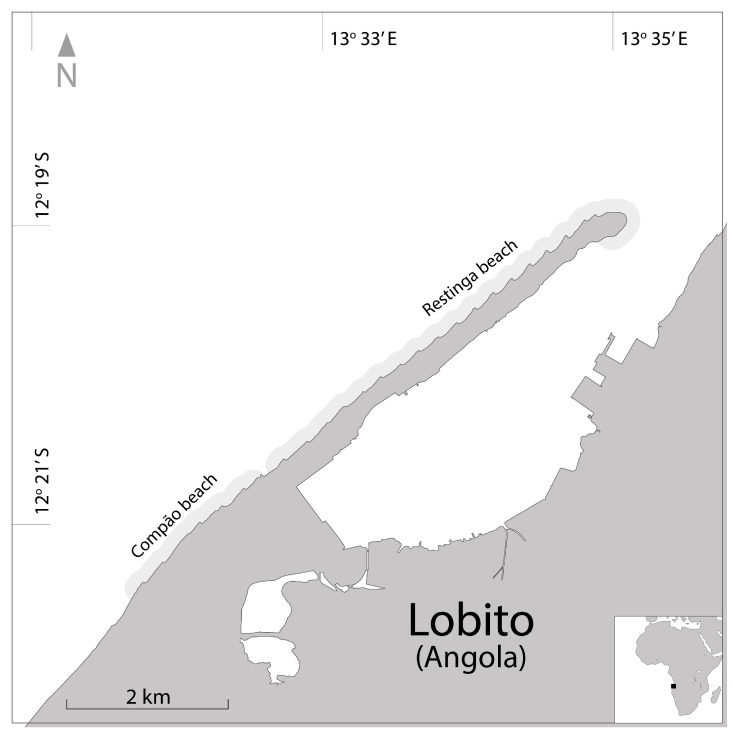
Restinga and Compão beaches context in the west coast of Africa (image created using adobe illustrator, trial version; credits: Cardoso, S.).

This project was conducted on the beaches of Restinga (12°19′37″ S 13°34′6″ E). It expanded in the 22–23 season to include monitoring on some beaches in Compão (12°21′24″ S 13°31′57″ E) in Lobito (Figure 1).

Lobito is a coastal city situated within the province of Benguela, located in the southern region of Angola. It is renowned for its thriving fishing industry associated with the Benguela Current, a cold oceanic current that originates by flowing northward along the western shoreline of southern Africa, extending from Cape Point in South Africa to Angola. This dynamic current plays a pivotal role contributing to the abundance of marine organisms, including sea turtles [38]. It is worth noting that the Benguela Current holds significance not just due to the nutrient input resulting from upwelling but also because it probably has an impact on hatchling dispersal towards the open sea. 

Restinga and Compão beaches show different geomorphological characteristics. Restinga is a geographic region characterized by elongated sandy deposits (with a length of approximately 4.8 km) that run parallel to the coastline, which are formed through sedimentation processes. Restinga is a semi-enclosed system comprising a bay and the open sea, featuring beaches that extend along its entire length, separated by spurs. Furthermore, it comprises urban, tourist, and industrial areas, as well as a port. The beaches of Compão feature extensive sandy areas that are located within the coastal zone of the city, which is an urban area under significant anthropogenic pressure due to the presence of many fishermen who perform continuous net fishing activities on these same beaches throughout the day. The sand of these beaches is of the siliceous type with a yellow color. The vegetation is scarce, and when present, it is found in the upper zone of the beach.

The patrol areas are divided by locality. In Restinga, the patrolled beaches range from Lighthouse Beach to Hotel Terminus Beach; in Compão, the patrols begin at Vila Mar Hotel Beach, which is immediately adjacent to Hotel Terminus Beach, and they extend to Feira Beach (Figure 1).

### 2.2. Field Effort

In the last three seasons (2020–2023), the field effort significantly increased, and it was more consistent when compared to the beginning of the project (2017), with the establishment of a fixed team of patrollers and the participation of volunteers.

In each locality (Restinga since the season 20–21; Compão since the season 22–23), a stable team of four patrollers was established who worked in pairs and alternated their workdays. These patrols also included volunteers whose numbers fluctuated based on the availability of each member, varying across different seasons, days, and hours.

The nesting season of the Olive Ridley species in Lobito occurs between the months of October and February, during which the field effort varied. In the second half of October and February, due to the lower occurrence of nesting females, only one person conducted a morning census for the potential identification of nesting activity. For the remainder of the season, the patrols occurred every night between 10 pm and 6 am, with a fixed team of 7 members (4 at Restinga in pairs and 3 at Compão). However, the team had members available during the day (24/7) to attend to any beach whenever necessary in case a female was nesting outside the patrol period.

Each patrol group carried with them one white flashlight, two measuring tapes, two buckets, five or more data recording sheets, and writing materials. Additionally, each patroller was individually equipped with red light headlamps and identification vests. The individual tagging materials, including PIT tags, reader, and camera, were solely the responsibility of a designated trained person responsible for tagging all nesting females.

### 2.3. Nests

All nests were relocated, becoming *ex situ*. The relocation of nests was conducted all through the breeding season. This measure had to do with high predatory pressure and human interference observed prior to the project, negatively impacting incubation. Translocation was therefore conducted as an effort to lessen this problem. There were two incubation sites: the boxes and the hatchery.

The boxes were made of wood lined with plastic and open at the top. The eggs were placed inside the box above a layer of sand and were subsequently covered with damp sand from the nesting site and left to incubate in a shaded area.

The hatchery was an enclosed and covered area; each nest was positioned at the center of a pre-marked 1 m^3^ area at a depth of approximately 55 cm, arranged to closely mimic a natural nest, and then covered with the same sand.

Some nesting parameters recorded and analyzed concerned oviposition (of the nests *in situ*, before taking the eggs to the *ex situ* places), clutch size, which corresponded to the number of eggs deposited in a single clutch by a specific female [39], and nest depth, which was determined by measuring the vertical distance from the point of initial egg deposition to the level of the sand using a tape measure [39].

The other parameters concerned the nests in *ex situ* places, the incubation period which corresponded to the period between the day of egg deposition and the day of hatchling birth [39], and hatching success, which was considered as the number of eggs that hatched [39].

### 2.4. Biometric Information

For each female turtle that was seen nesting, the date and time when the turtle was found in the locality and its respective beach were recorded. The measurement of the minimum curved carapace length (CCL) was conducted using a flexible measuring tape that had a length of 1.5 m and was calibrated in metric units. This measurement involved determining the distance between the anterior midline point, also known as the nuchal scute, and the posterior midline notch located between the supracaudals. The maximum width (CCW) was also documented in the measurement of the carapace width, which was performed at its maximum width without relying on any anatomical landmarks [40]. Irregularities in the carapace such as wear, parasites, predation marks, and deficiencies were recorded, as they could affect the measurements and could be used to individually identify nesting females.

### 2.5. PIT Tags

The project decision was made to utilize PIT tags for individual marking to mitigate the risks associated with infections and the attrition rates observed with metal tags.

PIT tags, which are microprocessors sealed in glass, transmit a unique identification number to a handheld chip ID scanner using a low-frequency radio signal over a short distance, and these were used to identify individuals.

PIT tagging began in the 22–23 season, and 30 nesting females were marked. The methodology for PIT tag placement followed the guidelines recommended by Balazs [41]. Initially, the patrols checked if there was any form of individual tagging (PIT, metal or plastic tags, or a transmitter) using an Agatige X001FOJRQB chip ID scanner (with the following specifications: reading standard: FDX-B (11784/5) and EMID; reading distance: 15 cm; working frequency: 134.2 kHz; certification: CE, ROHS), although there was no record of sea turtle tagging on the coast of Angola. Subsequently, a 2.12 × 12.00 mm PIT tag compliant with ISO 11784/11785 FDX-B standards and designed for animal identification provided in individual sterilized syringes was applied to the front right flipper under the skin of the second scale.

### 2.6. Photo Identification

The best photos of each individual were selected, from both the right- and left-face sides, as well as the carapace and head (together or separately). If any photograph was found to be of insufficient quality for subsequent analysis, the photographs were repeated. The camera used for this purpose was that of the mobile phone of the patrollers, and the model varied according to the availability of equipment. Subsequently, the photographs were cataloged, with each female identification (when possible, through the assignment of the PIT number) and each photograph categorized according to the area photographed, thus creating a database for further analysis.

### 2.7. Statistical Analysis

The analysis of the data regarding nesting ecology encompassed records from the years 2020 to 2023. 

Descriptive statistical analysis was conducted using the Microsoft^®^ Excel^®^ para Microsoft 365 MSO (version 2311 Build 16. 0. 17029. 20108) 32-bit, with significance estimated at a 95% confidence level. The Pearson correlation coefficient was used to examine the associations between female size variables, namely CCL and CCW. 

The results regarding CCL and nest depth were analyzed by one-way analysis of variance (ANOVA) repeated measures followed by Tukey’s post-hoc test in GraphPad Prism, version 9.5.1.; for all analyses, the significance level was set at 0.05.

## 3. Results

A total of 455 records were analyzed. Of these, 193 records corresponded to sightings of nesting females distributed as follows by nesting season: during the period of 20–21, a total of 62 females were sighted nesting; in the subsequent period of 21–22, the number of sights decreased to 57; however, in the period of 22–23, the number of sights increased to 74 females, out of which 30 were successfully tagged.

At least two of the tagged females (A and B) returned to nest again. Female A, tagged during her initial nesting on 18 November 2022, laid 141 eggs. She was sighted again on 27 December 2022 and laid 101 eggs (39 days between sightings). Female B nested on 10 December 2022 and laid 27 eggs, while the second observation was recorded on 20 December 2022 with a clutch size of 89 eggs (10 days between sightings). Additionally, there was a deceased female found, which was tagged on 19 November 2022 and discovered dead by a beach guard on 2 January 2023 (45 days between sightings). Upon receiving information regarding the turtle’s location from the guard, the patrollers proceeded to verify that there were no indications of an attempted act of killing. However, observations revealed that an individual had immobilized the turtle using stones. An upward crawl track on the sand was observed, allowing for the identification of the turtle and confirming that no nest had been laid.

**Figure 2:**
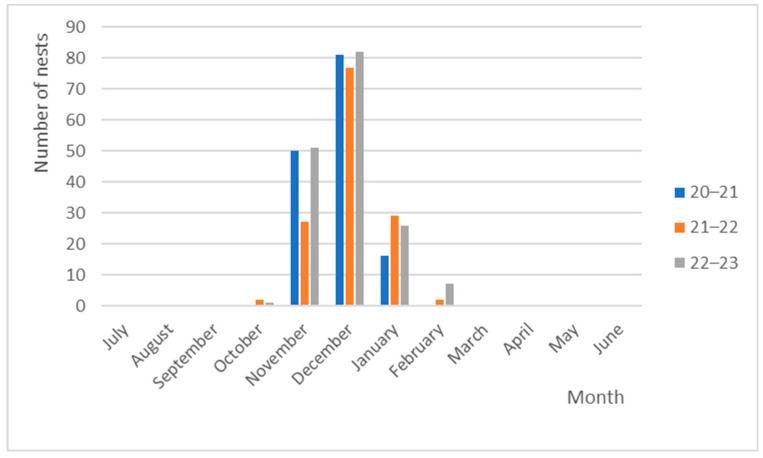
Distribution of the number of nests throughout the analyzed seasons.

By examining the quantities of nests and their corresponding dates, it is evident that December showed the highest frequency of nesting among the three seasons investigated, with a noteworthy total of 240 documented nests. In contrast, the month of October exhibited the lowest occurrence rate, with a mere three nests observed throughout the three seasons under analysis. In 20–21, no nests were documented, while 21–22 saw two nests, and 22–23 recorded a sole nest (Figure 2).

**Figure 3:**
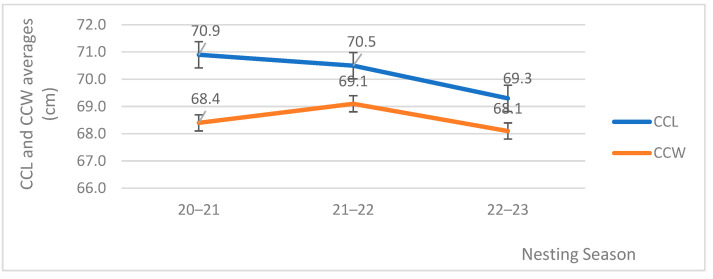
Average CCL and CCW of nesting females (*n* = 200) during each nesting season with tendency lines.

**Figure 4:**
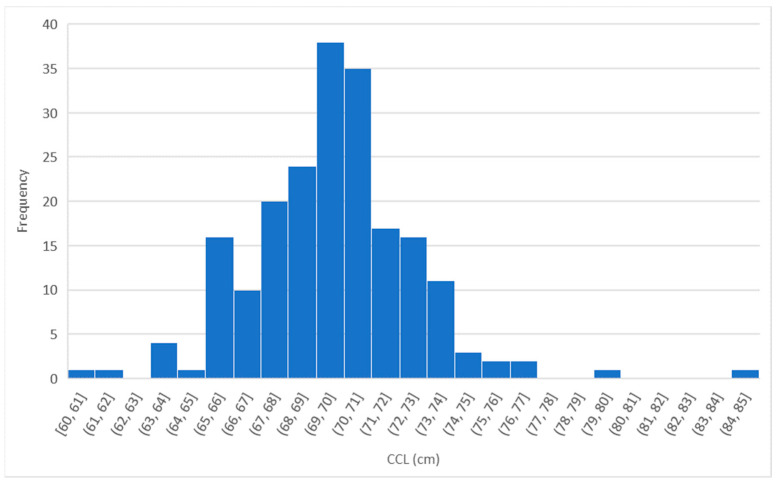
Distribution graph of minimum curved length, CCL (*n* = 200).

Over the study period, a total of 200 turtles were measured. The average CCL was 70.2 ± 0.2 cm (range 60.0–85.0 cm; Figure 3 and Figure 4) and the average CCW was 68.5 ± 0.2 cm (range 44.0–80.0 cm; Figure 3). The Pearson correlation coefficient (r) between CCL and CCW was 0.56, suggesting a moderate positive correlation. This means that there was a positive relationship between the two variables, and the strength of the correlation was moderate. The equation of the line describing this relationship was: y = 0.6212x + 24.926. This suggests that there was a relationship between CCL and CCW, but it was not a perfect or complete relationship, as indicated by the R^2^ value of 0.3158.

The averages of the carapace measures can be viewed in Figure 3, and the tendency lines show a decrease in these parameters over time. The CCL values decreased over the course of the study period. Regarding the CCW, there was also a tendency for the values to decrease over time, but in the 21–22 season, the average (69.1 cm) was higher than that of the following season (68.1 cm).

Through the analysis of Figure 4, it is possible to observe that the most represented CCL values were within the range of 69–70 cm.

**Figure 5:**
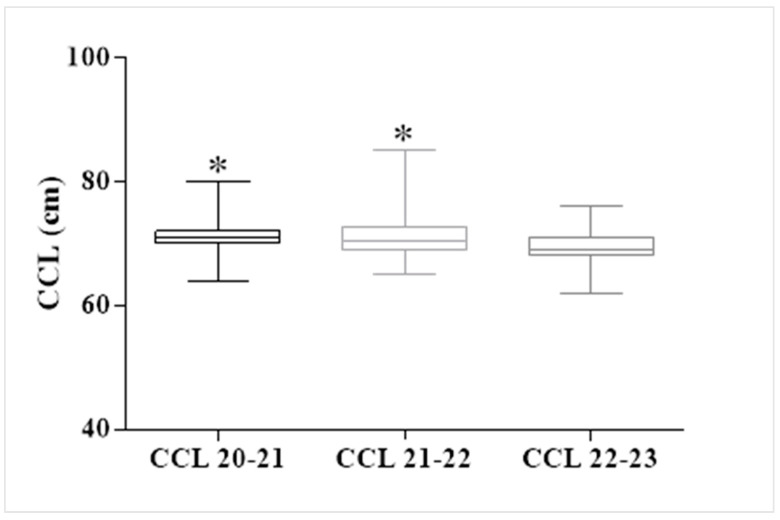
Results of repeated measures of analysis of variance (ANOVA) with Tukey’s post-hoc test for CCL between the seasons 20–21, 21–22, and 22–23. * indicates significant difference from corresponding values in the season 22–23 (*p* < 0.05).

To analyze differences in CCL across the various seasons, we conducted an ANOVA test, yielding an F-value of 6.83 and a *p*-value of 0.001, indicating the presence of differences among the groups. Therefore, we performed a multiple comparisons test (Tukey’s post hoc test) to make specific pairwise comparisons among the groups. This analysis revealed significant differences between the 20–21 and 21–22 seasons when compared to the 22–23 season (Figure 5). 

**Figure 6:**
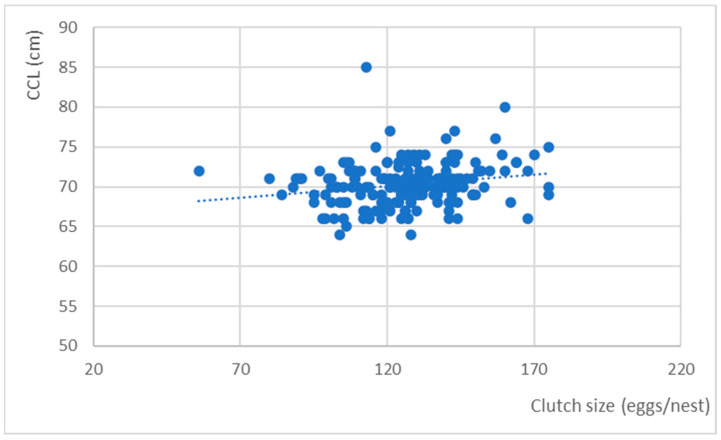
Correlation between the size (CCL) of nesting females and the number of eggs laid (clutch size) with the trendline.

A sub-sample (*n* = 186) was considered, in which partial clutches (fewer than 20 eggs) and the corresponding females that laid them were excluded, and only records in which both the CCL and the number of eggs were recorded were considered. A cluster around 70 cm for CCL was revealed, corresponding to clutches ranging from 100–150 eggs. The presented data are in clusters, but there was evidence of large females with both large and small clutches (although in a smaller percentage) and small females with both large and small clutches (Figure 6).

The average clutch size for the entire sample (*n* = 433) was 127 eggs/nest (range 56–175 eggs/nest; Table 1). Table 1 displays the means for each breeding season. The season 20–21 was the one with the lowest average clutch size, while the season 21–22 was the season with the highest average clutch size.

The clutches, from the three analyzed seasons, were similar, as the ANOVA test showed that there were no statistically relevant/significant differences (F-value was 2.05 and *p*-value was 0.13).

The mean incubation period for the season of 22–23 was 60 ± 1 days (Mo = 57 days), ranging from 32 to 90 days (*n* = 124). 

The *ex situ* hatching success rate was on average 82%, with the 22–23 season exhibiting the highest mean at 87%. 

The nests had an average depth of 47.0 ± 0.5 cm, with Mo = 50 cm (range: 9.3–79.0 cm). Based on the calculated F-value of 27.84 and the *p*-value lower than 0.001, it can be concluded that there were statistically significant differences between the seasons. Tukey’s post hoc test revealed statistically significant differences between the values seen in the 20–21 and 21–22 seasons compared to the corresponding values in the season 22–23. Additionally, there were statistically significant differences between the values observed in the 20–21 season and the corresponding values in the season 21–22.

**Figure 7:**
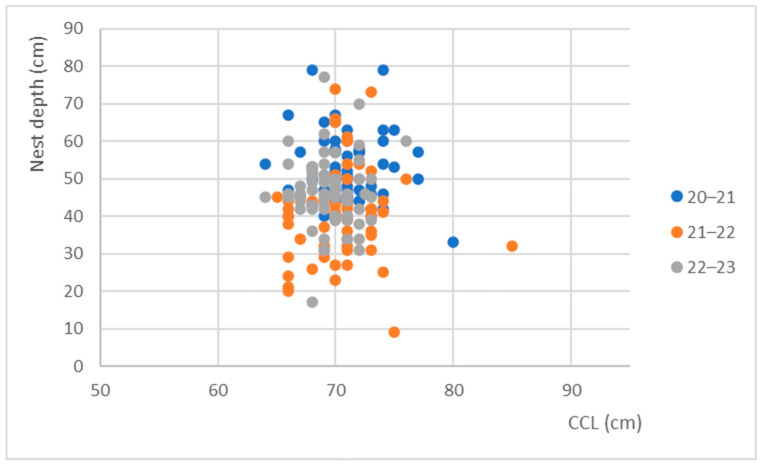
Relationship between nest depth and nesting female size across the seasons.

The data collected during the 20–21 season indicate that the nesting depth was significantly deeper, with an average of 51.5 cm, and it exhibited a smaller and deeper range of values (ranging from 23 cm to 79 cm) in comparison to the nesting depths found in following seasons. By analyzing Figure 7, it is possible to observe a shift in the cluster for each season with respect to the nest depth.

**Figure 8:**
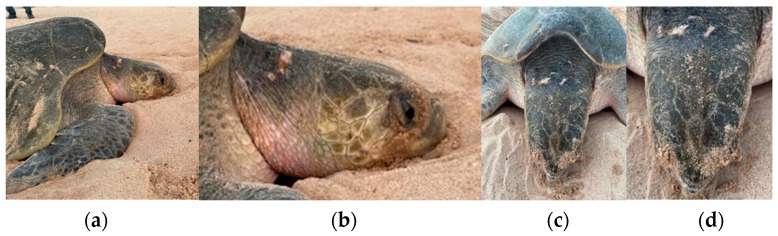
Example of photographs from a nesting female for individual analysis and identification: (**a**) right lateral view, (**b**) zoomed-in right lateral view, (**c**) top view of the head, and (**d**) zoomed-in top view of the head.

In the 22–23 season, 78 photographs of the 30 tagged turtles were collected. Figure 8 displays pictures of the lateral and superior views of an Olive Ridley nesting identified by the PIT tag number 900215003691608.

## 4. Discussion

### 4.1. Nesting Season

The nesting season of Olive Ridley examined in this study aligns with that described by other authors for different locations, i.e., the central/southern zone of Angola [15], Togo [42], and Brazil [43] (at the same latitude as Angola). It occurs during the same time frame (November–February), with occasional records in October and March. However, at higher latitudes, there is a shift in the nesting period: northeastern Brazil: March-August [44], Colombia: August-September [45], Australia: February-November [46], and India: December-April [16]. Despite all these locations falling within tropical zones, the selection of nesting sites by female turtles is influenced by a combination of variables encompassing temperature, meteorological conditions, physical attributes of the nesting beaches, the adjacent oceanic environment, tidal conditions, and patterns of temperature and surface currents [47,48]. Collectively, these factors contribute to the decision-making process employed by female turtles in determining their egg-laying locations [16].

### 4.2. Female Size and Clutches

*L. olivacea* exhibits a distinctive nesting behavior known as “arribada”, yet within the study area, this species reproduced in a solitary/dispersed manner, in line with findings from other researchers [14,15,49]. Also, the nesting females from this population had a distinguished behavior resembling a dance as they covered the nest, utilizing their back flippers to press the sand and tapping their plastron to firmly bind the sand around the nest.

Differences were observed between arribada-nesting and solitary-nesting females within the same population, with solitary females displaying a smaller average carapace size [16]. The average curved carapace length (CCL) of nesting females at Lobito was 70.2 ± 0.2 cm (range: 62 to 85 cm). This value surpassed that indicated by Tripathy [16] in Orissa for both solitary nesters (CCL = 66.02 cm) and arribada nesters (CCL = 67.16 cm). However, it was lower compared to females measured in Oman, with an average size of 74.1 cm [50]. Lobito’s values aligned with those reported by Reichart [51] for adults (55 cm or more), with those by Whiting et al. [46] for nesting *L. olivacea* in Australia/southeast Asia, with a mean CCL of 69.6 ± 2.3 cm (range: 65.0 to 75.2 cm, *n* = 85), and with those by Santos et al. [52], with sizes varying between 64–79 cm. 

The larger average size of females recorded in the three seasons (Figure 3) suggests an older population compared to other locations [53]. The origin of the breeding females in the Lobito area has remained unknown to date. The recruitment of individuals from northern regions may occur for diverse motives; however, the absence of pertinent information precludes the establishment of their residency or migration patterns. The possibility of accomplishing this task is contingent upon the implementation of genetic analyses, tracking device deployments, or a comprehensive tag study. Due to the limited timeframe of the project’s study period, it is premature to engage in conjecture regarding the factors contributing to the observed disparities in size among these individuals. The absence of information regarding the population’s background presents a significant obstacle in accurately ascribing the factors responsible for the variations in size. Nonetheless, the sporadic occurrence of smaller females could be a consequence of climate change [54] and/or anthropogenic pressures, leading to adaptive changes and the recruitment of smaller nesting females [55] from other territories (Sierra Leone? Liberia? Ghana? Gabon? Republic of Congo?). The analysis and discussion of these parameters are essential for population characterization and trend assessment, as they are interrelated [55,56,57,58,59,60]. It is important to acknowledge that the temporal scope of sampling in this study was limited, and there exists uncertainty in terms of the duration between successive nesting periods. Furthermore, it is necessary to acknowledge the influence of climate change on the behavior of this species. 

The mean clutch size for Lobito’s female *L. olivacea* was 127 eggs per nest, with Mo = 128 eggs per nest. These values surpassed the values given by Pritchard and Plotkin [61], where approximately 100–110 eggs per clutch were observed for individual Olive Ridley females. These values were even higher when compared to nesting occurrences at El Valle beach, where the average egg count per nest was 87 ± 14 [45].

Regarding the nest depth, a variation in the nest depth between seasons was evident. In the 20–21 season, the nests exhibited a greater depth (51.5 cm); in all seasons, but more prominently during the period of 21–22, it was possible to verify the existence of some nests that were no deeper than 30 cm. This means that, in cases when the clutches contained more than 100 eggs, the eggs were near the surface and therefore more susceptible to fluctuations in the weather. However, there was no observed correlation between the female size and nest depth in this analysis, as said by other authors [62]. This suggests that the depth of the nests cannot be entirely described by the size of the females [62]. As described, various abiotic and biotic factors influence nesting site selection by females [47,63]. The choice of a nesting location based solely on surface temperature and humidity was previously discarded due to rapid variability [47]. However, instances of turtles abandoning nesting attempts due to hard sediment or encountering rocky substrates were observed. This raises the hypothesis that, besides surface conditions, depth-related parameters influence nesting choices. This prompts the question of how nesting females perceive conditions within the nest during excavation. Is there a capacity, perhaps through flippers, to assess temperature/humidity conditions? What factors drive variable nesting depths? Given their perception of abiotic conditions and the critical role of temperature and humidity in hatchling success, it is plausible that these turtles possess attributes enabling the assessment of such parameters within the nest, especially as some researchers propose their selection of optimal nesting locations [47,48]. Although there is no defined pattern yet, it has been questioned whether this could be a behavior of the species in response to climate change, demonstrating the species’ capacity for behavioral plasticity in response to local conditions [62]. Consequently, in the 20–21 season, a deeper excavation likely occurred in search of these conditions, with deeper nests exhibiting, probably, lower, and more consistent average temperatures [64,65]. 

### 4.3. PIT Tags

The application of PIT tags represents an effective tool for individual identification, consequently contributing to the determination of population and/or subpopulation sizes [40,66]. Nevertheless, the duration of the observation period, which was limited to a single breeding season, and the small sample size of 30 individuals used in this study were inadequate for reliably evaluating the nesting population size of the region. Hence, it is imperative to continue the tagging to carry out possible future assessments of population numbers.

Similarly, the assessment of inter-nesting periods for nesting females remains inconclusive due to the recapture of only three out of the thirty marked females. The intervals between captures were 10, 39, and 45 days. This could indicate that the inter-nesting interval is approximately 10–15 days, considering that within the 39th to 45th day interval, the recaptured females laid two additional nests that were not identified. These low recapture figures are linked to the limited field effort conducted during the season. However, the data aligns with the interval range (9–23 days) reported by Santos et al. [52] in Brazil. 

Conclusive inferences regarding the nesting population will only become viable with an expansion in terms of the sampling period, sample size, and field effort. A population size analysis is imperative for devising effective mitigation measures tailored to a specific population [67,68].

The use of PIT tags offers several advantages [66], causing reduced pain to the animals, a lower susceptibility to infections, and a nearly negligible identity loss [41,69]. However, the application of this methodology requires skilled and trained labor for its execution [70,71]. There is also the cost associated with PITs [72,73], which are more expensive and require handheld scanners, and all groups in the area need to possess handheld scanners to identify individuals. In comparison, flipper tags are easily readable by other groups, fishermen, and the general public, while PIT tags restrict the future recording of data beyond the nesting beach [74]. Additionally, access to such materials is challenging and sometimes unavailable.

### 4.4. Photo Identification

The utilization of photographs for individual identification is a tool that is considered advantageous [75] due to its non-invasiveness, stress reduction, and pain alleviation [76] as opposed to invasive tagging methods [77]. Coupled with available artificial intelligence, several studies have been conducted to explore the fidelity of these tools and their capacity to accurately identify individuals during mark–recapture events [78].

However, these studies primarily focus on individuals in marine environments [79,80] rather than nesting females. One of the challenges in this type of identification is the quality of photographs [81]. For nesting females, the substantial amount of sand often hampers the proper identification of scale in photographs [74] and so frequently requires manual cleaning, introducing human intervention and diminishing the method’s advantages. Additionally, lighting poses a problem, as nesting occurs at night, requiring artificial illumination for analyzable photographs. Considering the photosensitivity of these species [82,83], the use of white artificial light for photography (facial and carapace features in this study) once again renders this method disadvantageous. However, in an attempt to mitigate the stressor of light (on faces), the photography of flippers could be considered, as tested by Gatto et al. [84] and Oliveira et al. [85]. Yet, the sand-associated issue persists, as these studies were conducted in marine, not terrestrial, environments. In the present study, the photographs (*n* = 78) of 30 nesting females were found to lack enough quality for a comprehensive analysis due to factors such as sand interference, lighting conditions, and subject movement. 

Nevertheless, in small-scale projects such as the Cambeú Project with limited funding or with a workforce not adept at employing other identification methods, photo identification could serve as an alternative to attempting to identify nesting females and tally the population count. It should be noted, however, that this method alone, for identifying nesting females on the beach, presents all the difficulties and disadvantages that have been previously mentioned.

### 4.5. Conservation Measures and Lessons Learned

In accordance with Eckert et al. [86], the primary objective of a conservation initiative is to enhance the long-term viability of sea turtle populations. To accomplish this goal, the Cambeú Project has implemented a framework to facilitate its advancement. As a result, the project has developed strategies aimed at preserving nesting beaches, ensuring the safety of nesting females, and relocating nests to more secure locations. Meanwhile, a community-based conservation approach has been developed, which involves the active participation of multiple sectors. This entails providing community training, coordinating recreational activities, involving influential community leaders, and cultivating collaborations with nearby hotels and restaurants, all with the aim of attaining equitable outcomes. The interconnectedness of the environmental and human groups is acknowledged, as is respect for cultural behaviors and traditions that do not harm sea turtles. 

The Cambeú Project places a high priority on the preservation of the environment and the use of resources in a sustainable manner, considering economic, social, and ecological considerations. The objective is to minimize socioeconomic expenditures and disputes while establishing a designated and safeguarded region [87]. The primary objective of this initiative is to implement conservation strategies to mitigate the loss in Olive Ridley populations in Lobito and its surrounding areas, along with other species of sea turtles. This project employs a conservation system that relies on community participation, aiming to ensure the provision of accurate and reliable data. This approach requires a shift in behaviors and perceptions [88]. It has (the Cambeú Project) experienced growth attributable to the voluntary contributions of community members and financial support from local establishments, including coastal restaurants and hotels. However, the required adequate labor, funding, and comprehensive knowledge regarding the local population and its dynamics, along with pertinent laws and regulations, are indispensable for ensuring that the project operates efficiently and in congruence with social and biological objectives.

It is essential that standard methodologies are followed rigorously. Nevertheless, it is worth considering the exploration of novel tools that have the potential to enhance fieldwork, not only to this project but on a global scale. Recent years have witnessed notable technological innovations, leading to a growing range of electronic gadgets that have demonstrated their effectiveness as supportive elements in conservation approaches. The application of unmanned aerial vehicles (UAVs), often known as drones, has been employed in the realm of marine turtle conservation to investigate the population size and behavioral patterns of these species. This technological approach has been effective in minimizing the need for labor-intensive field work, as evidenced by studies conducted by Schofield et al. [89] and Sellés-Rios et al. [90]. 

Another prospective strategy for enhancing field operations and increasing patrol effectiveness could entail the deployment of dogs, relying on their extensively refined olfactory capabilities. Although dogs have been recognized as predators of turtle nests [91], they possess a notable ability to learn [92]. The development of skills in canines is contingent upon their breed, and the process of training necessitates adherence to the three stages of canine training: habituation, sensitization, and operant conditioning [93]. This approach proves to be particularly valuable in scenarios where patrols may not have the opportunity to directly observe the nesting behavior of female turtles but instead find the nest after it has been laid. In situations of this kind, the task of locating the nest can prove to be challenging, as the nesting females frequently employ a substantial camouflage process, thereby delaying the ability of human patrols to successfully locate it. In order to optimize the ability of canines to locate nests without causing any detrimental effects, it is crucial to administer tailored and precise training protocols.

The parameters studied in this work include notably short incubation periods in the hatchery, which can be an indicator of high temperatures [94]. If that is the case, the atmospheric and environmental temperatures in this area may surpass the species’ optimal survival conditions, which may result in short-term consequences for the population. Hence, in the absence of intervention, these alterations could potentially lead to the extinction of the nesting habitat. Thus, the Cambeú Project favors *ex situ* incubation, since it has shown the most successful results and might represent the only viable approach to ensuring the survival of hatchlings in the Lobito region. 

The importance of this matter lies in the potential consequences of the species’ local extinction on the surrounding community once it is evident that the existence of this species has already had effects on the local economy. The project directly employs several collaborators, both part-time and full-time, and is sought after by new young volunteers each year who wish to contribute to the project. It also contributes to local tourism by establishing partnerships with various coastal hotels and restaurants where the hatchlings are released into the sea. This conservation action serves as a moment of environmental awareness and attracts visitors from outside the region who come to explore the project’s beaches. Additionally, the project collaborates with schools, welcoming approximately 1440 children annually to its headquarters for an introduction to turtle conservation through engaging educational activities. A wide range of lectures are also offered to teachers and university students. Therefore, the extinction of this nesting ground, as hypothesized, might potentially result in economic repercussions for the local people.

### 4.6. Future

In a complex region spanning from Mauritania to the Democratic Republic of Congo, where the coast is shared by multiple countries, knowledge is limited. In the southern part, only Angola and Namibia are present. Despite the scarcity of information, the shared oceanic life, migratory routes, and distribution of different populations remain largely unexplored [95]. The conservation of marine biodiversity in the context of Atlantic Africa poses a significant challenge for the countries involved.

Despite the numerous admonitions highlighting the imperative nature of studying the African coastline, it remains essential to reiterate and reinforce this exigent requirement. Having comprehensive data to demarcate geographical zones of habitation is of paramount importance, enabling the exploration and establishment of connections between *L. olivacea* territories and the boundaries of other populations. However, this endeavor is hindered by a lack of accessible information. A synergetic effort must emerge to gather consolidated ecological, demographic, and molecular data of each marine turtle population, or even other indicators concerning the connectivity between territories used by marine turtles. Combining tracking techniques with genetic analysis would be of significance, as it would unveil the migratory pathways of the population and the interconnectivity with the other RMUs, helping establish areas of occupancy and addressing information gaps. This approach would facilitate the implementation of efficacious mitigation measures, not only at national/regional levels but also on an international scale, thereby fostering international cooperation. The current fieldwork primarily focuses on monitoring nesting beaches and translocating nests, providing valuable insights and a promising conservation outlook. It demonstrates that with increased funding, this conservation strategy can be effectively implemented along the west African coast where it is currently underrepresented, and further improvements are achievable.

## Figures and Tables

**Table 1:** Average clutch size, nest depth, incubation period, and hatching success over the seasons (2020–2023).

Averages	20–21	21–22	22–23	Total
Clutch size (eggs/nest)	124 ± 1 (*n* = 145)	129 ± 2 (*n* = 136)	127 ± 1 (*n* = 152)	127 ± 1 (*n* = 433)
Nest depth (cm)	51.5 ± 0.7 (*n* = 143)	42.4 ± 1.1 (*n* = 136)	46.6 ± 0.8 (*n* = 143)	47.0 ± 0.5 (*n* = 422)
Incubation period (days)	-	-	60 ± 1 (*n* = 124)	60 ± 1 (*n* = 124)
Hatching success (%)	83 ± 2 (*n* = 146)	75 ± 2 (*n* = 137)	87 ± 1 (*n* = 165)	82 ± 1 (*n* = 448)

## Data Availability

The data can be provided when requested.
